# Effectiveness of the self-efficacy modification program for Thai alcoholic inpatients with schizophrenia

**DOI:** 10.1007/s44192-025-00296-1

**Published:** 2025-10-21

**Authors:** Tirapada Kiatsangorn, Doungjai Polngam, Nimit Kaewarj, Benjayamas Pilayon, Wuttiphong Phakdeekul, Nitikorn Phoosuwan

**Affiliations:** 1 Nakhon Phanom Rajanagarindra Psychiatric Hospital, Nakhon Phanom, Thailand; 2 Khon Kaen Rajanagarindra Psychiatric Hospital, Khon Kaen, Thailand; 3 Boromarajonani College of Nursing Nakhon Phanom, Nakhon Phanom, Thailand; 4 Department of Public Health Administration, Kasetsart University Chalermphrakiat Sakon Nakhon Province Campus, Sakon Nakhon, Thailand; 5 Faculty of Public Health, Thammasat University, Pathum Thani, Thailand; 6 Department of Public Health and Caring Sciences, Faculty of Medicine, Uppsala University, Uppsala, Sweden

**Keywords:** Alcohol use disorder, Comorbidity, Inpatients, Schizophrenia, Self-efficacy, SEMASchizo program, Thailand

## Abstract

**Background:**

In response to the evident limitations of conventional treatment approaches and the consequent recurrence of alcoholic people admitted with schizophrenia behavior leading to re-hospitalization, a program was instituted at a psychiatric hospital in Thailand. This program aimed to modify alcohol consumption behavior patients undergoing inpatient treatment by incorporating the principles of Bandura’s Self-Efficacy Theory.

**Method:**

In this two-group quasi-experimental study, the impact of the Self-efficacy modification program was investigated among alcoholic people admitted with schizophrenia. This study involved two sample groups: an intervention and a control group, each consisting of 32 participants. The intervention group underwent a 5-week intervention featuring 14 group therapy activities, where the control group received a usual treatment. Data analysis employed descriptive statistical methods and involved comprehensive questionnaire data collection across four dimensions i.e. Stages of change readiness and treatment eagerness scale (Socrates-8 A), Severity of alcohol dependence questionnaire (SADQ), Alcohol abstinence self-efficacy scale (AASE), Brief psychiatric rating scale (BPRS). Data were analyzed using independent and paired samples t-tests, with a significant level of 0.05.

**Results:**

The paired t-tests conducted on changes in Socrates 8 A and AASE scores before and after the intervention program demonstrated a significant increase in scores, coupled with a decrease in BPRS scores, with all findings indicating a statistically significant difference (p-value < 0.001).

**Conclusion:**

This modification program could serve as an alternative intervention for alcoholic people admitted with schizophrenia. Policymakers should support training initiatives to equip mental health professionals with the skills needed to enhance self-efficacy in alcoholic patients with schizophrenia.

## Introduction

Schizophrenia ranked among the top 20 serious diseases worldwide, affects approximately 1% of the world’s population [1]. This elevates the risk of consuming large amounts of alcohol to three times higher than the general population [2]. The prevalence of lifetime alcohol use disorder (AUD) is reported at 24.3%, with a study indicating that 36.4% have experienced AUD before the first onset of schizophrenia, and a majority of these cases are found among males [3]. Individuals with schizophrenia and alcohol use problems face an elevated risk of depression, suicide, and aggression, coupled with the necessity of regular medication intake. Additionally, they contend with chronic physical health problems and experience high rates of hospital admissions [4]. According to World Health Organization (WHO) data from 2018, over 3.1 billion people globally, constituting 57.0% of the population aged 15 years and over, engage in alcohol consumption. The average person currently drinks 6.4 L per year, with predictions indicating an increase to 7.0 L per person annually by the year 2025 [5]. Thailand ranks among the top five in alcohol consumption in Southeast Asia, with the age group of 25–44 years old representing the highest percentage at 35.4% [6]. In the year 2020, study results revealed that doctors diagnosed 6.3% of the Thai population as alcohol addicts [7].

The 2020 National Epidemiological Survey of Mental Health in Thailand discovered that alcohol-drinking behavior disorders exhibited the highest prevalence, with a lifetime prevalence of 18.0% and a 12-month prevalence of 5.3% among individuals with psychiatric disorders. Moreover, those with alcohol-drinking behavior disorders had limited access to services, with only 6.6% seeking assistance [8].

In 2021, a psychiatric hospital in Thailand, equipped with 90 beds, admitted 87 alcoholic people diagnosed with schizophrenia for inpatient treatment. Of these, 31 individuals, constituting 35.6%, returned for repeat treatment. In 2022, the hospital admitted 109 cases with the same dual diagnosis, and 47 of them sought repeat treatment, accounting for 43.1% of the total cases [9]. Despite adherence to current care guidelines, there has been a lack of significant changes in patient behavior, leading to recurrent hospital readmissions. Notably, the most prevalent causes for readmission are identified as a combination of non-compliance with medication and concurrent alcohol use. Moreover, the lack of studies on co-occurring alcohol use and schizophrenia complicates clinical management for mental health professionals. In response, the researcher has initiated a program designed to modify alcohol consumption behaviors, drawing inspiration and refinement from Kaewarj et al. [10]. This program is structured according to the principles of the self-efficacy theory [11], aiming to instill confidence in individuals to successfully execute behavior aligned with their established goals. After completing the program, alcoholic inpatients with schizophrenia may demonstrate increased treatment readiness, reduced alcohol dependence, fewer psychiatric symptoms, and greater self-efficacy in maintaining abstinence. The anticipated outcome is a reduction in alcohol consumption among alcoholic people diagnosed with schizophrenia, ultimately enhancing the efficacy of the treatment. The success of this study may pave the way for broader implementation of the findings in diverse healthcare settings.

## Materials and methods

### Aim

This study aimed to evaluate readiness to change and the desire for treatment of alcohol dependence, alcohol dependence severity, self-efficacy in quitting alcohol, and psychological symptoms among alcoholic people admitted with schizophrenia at one psychiatric hospital in Thailand after participating in the self-efficacy modification program.

### Study design and setting

Using a quasi-experimental design, two-group quasi-experimental study pre-test and post-test design. Data were collected from the inpatient department for 5 months (October 2023 – February 2024) from one psychiatric hospital in Thailand.

### Sample size and sampling

Participants were selected using consecutive sampling. The sample size for the study was determined using a formula designed for testing hypotheses, specifically for assessing the difference in means between two independent groups. The study utilized pooled variance obtained from a literature review from Kaewarj et al. [10]. Based on the calculated pool variance of 46.15 and effect size (difference in means) of 5.31 (µ1 = 11.27 for the control group, µ2 = 5.96 for the intervention group), a 95% confidence interval was set with α = 0.05 and Zα = 1.96. To achieve 80% test power with β = 0.20 and Zβ = 0.84, the sample size was determined to be 25.66 cases. To compensate for a possible 20% loss in the follow-up, it was decided to include 32 participants per group.

The inclusion criteria consist of alcoholic people diagnosed with schizophrenia based on the International Classification of Diseases, Tenth Revision (ICD-10) criteria (F20.xx for schizophrenia and F10.xx for alcohol-related disorders) [12], aged 18 to 60 years, who had a history of dangerous drinking (scored between 8 and 19 on the Alcohol Use Disorders Identification Test: AUDIT) in the past year and exhibited symptom relief (with Brief Psychiatric Rating Scale: BPRS scores decreasing by at least 24% or not exceeding 36 points), were capable of effective communication and cognitive function (Thai Mental State Examination: TMSE score ≥ 26) and scored 0 on the Alcohol Withdrawal Scale (AWS). Whereas, participants who had recurrent psychotic symptoms, violent and aggressive behavior, or were unable to follow instructions were excluded from the study.

### Self-Efficacy modification program among alcoholic people admitted with schizophrenia (SEMASchizo program)

The researcher has incorporated [13] a program from Kaewarj et al. [10]. This program is a revised version of motivational activities developed and enhanced by Wittayathawornwong et al. [14]. The program was evaluated for suitability by three experts: a psychiatric nurse, a behavioral science instructor, and a physician. The results indicated an availability of suitability, with an average score of 0.9–1.0 (Index of item objective congruence: IOC). A score in the range of 0.5–1.0 has acceptable accuracy. The researchers (TK and DP) seasoned a registered nurse and a professional pharmacist regularly work in the psychiatric ward of the specified hospital. Possessing extensive expertise in psychosocial care and therapy for psychiatric patients, they have undergone specialized training in mental health and psychiatric nursing through the department of mental health. The program consisted of 14 group therapy activities delivered over 5 weeks, each of which is aligned with the principles of self-efficacy theory to achieve the desired outcomes.

**Fig. 1:**
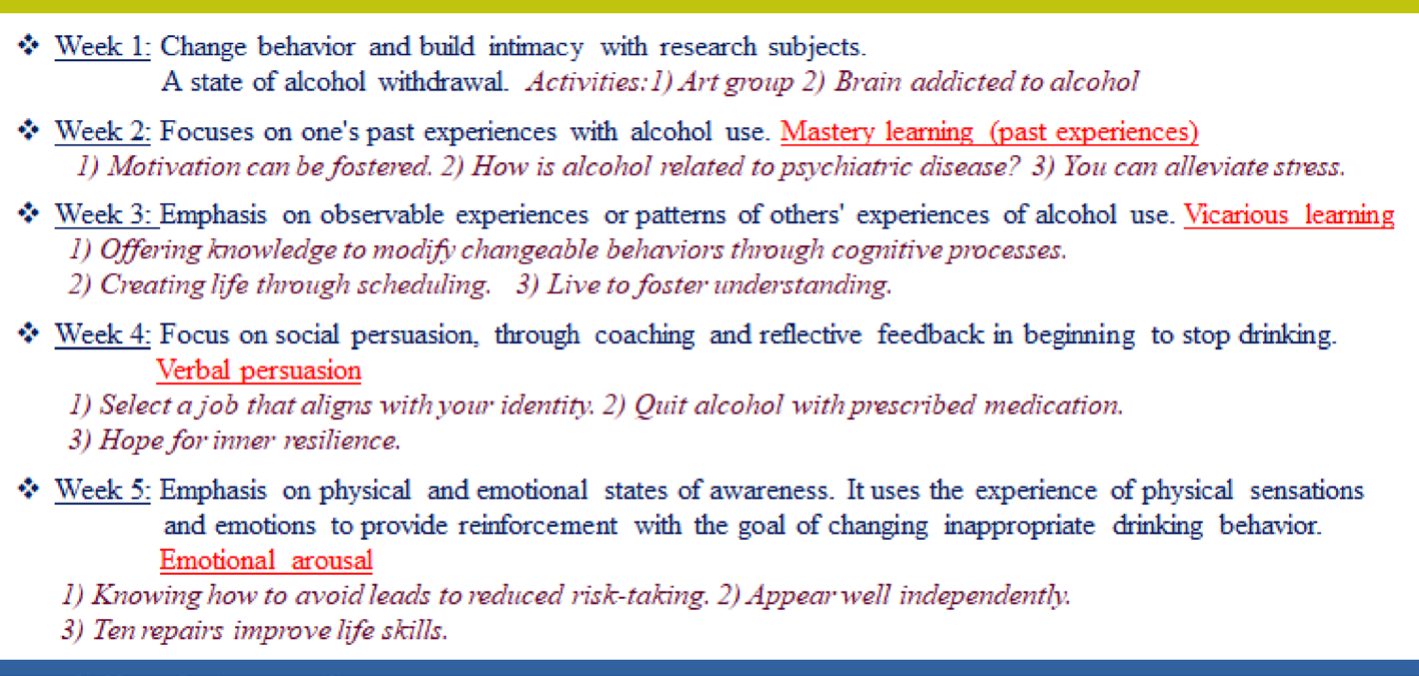
The self-efficacy modification program among alcoholic people admitted with schizophrenia at one psychiatric Hospital in Thailand

The SEMASchizo program spans a 5-week for inpatients. Each activity occurs with 2 days between them, and participation eligibility is determined by the activity organizer, aligning with the four conceptual components of self-efficacy theory. During the program implementation within the ward, counseling sessions are conducted in dedicated patient rooms, ensuring a group size limited to eight patients to safeguard the well-being of other psychiatric patients. Throughout the study period, a total of 32 individuals were divided into four groups, each comprising eight people, and they underwent inpatient treatment during various periods. See Fig. 1.

### Instruments

The tools employed in this study included a demographic questionnaire (participant characteristics), the SEMASchizo program, and comprehensive questionnaire data collected across four domains: stage of change readiness, severity of alcohol dependence, self-efficacy, and psychiatric symptom rating. The revised questionnaire was reviewed by three experts to assess its accuracy, with a focus on content validity and coverage, resulting in IOC values ranging from 0.9 to 1.0. Subsequently, a tryout was conducted among 30 individuals with similar characteristics to determine the reliability of the instrument. Overall Cronbach’s alpha coefficient was 0.923, indicating high reliability.

The Stages of Change Readiness and Treatment Eagerness Scale (SOCRATES-8 A for AUD), developed by Miller and Tonigan [15] and adapted into a Thai version [16], was employed. The instrument consists of 19 items assessing three domains: Recognition (7 items), Ambivalence (4 items), and Taking Steps (8 items). Each item is rated on a 5-point scale, yielding a total score range of 19–90. The Severity of Alcohol Dependence Questionnaire (SADQ), Thai-validated version [17] adapted from the original instrument [18], was also utilized. It comprises 20 items, each rated on a 4-point scale (0–3), with a total possible score of 0–60.

The Alcohol Abstinence Self-Efficacy Scale (AASE) [19], Thai-translated version [20], was employed. It comprises 20 items, each rated on a 5-point scale, yielding a total score range of 20–100. The Brief Psychiatric Rating Scale (BPRS) [21], Thai-validated version [16], was also utilized. This instrument consists of 18 items, each rated on a 7-point scale, with a total possible score of 18–90.

The Cronbach’s alpha coefficients for the Socrates-8 A for AUD, SADQ, AASE, and BPRS were 0.776, 0.837, 0.849, and 0.839, respectively.

### Procedure

After receiving approval from the Human Research Ethics Committee, the principal investigator (TK) obtained permission from the hospital director to conduct the study and collect data. Subsequently, the research team (TK, DP, and NP) assessed the eligibility of potential participants. Eligible individuals were invited to participate through an official invitation letter and a structured baseline questionnaire (T1) designed to collect demographic information and study-related details. An informed consent form was provided, accompanied by written information explaining the study’s purpose. It was clearly stated that participation was voluntary and that participants retained the right to withdraw at any time. All 64 invited participants (*n* = 32 in the intervention group and *n* = 32 in the control group) signed the consent form. The intervention group participated in the SEMASchizo program, while the control group received standard inpatient care, which included medication management, supportive counseling, psychoeducation, and occupational therapy in accordance with the hospital’s routine practices [22, 23]. As all participants were inpatients with schizophrenia, they were prescribed antipsychotic medications consistent with standard inpatient treatment protocols [24, 25].

**Fig. 2:**
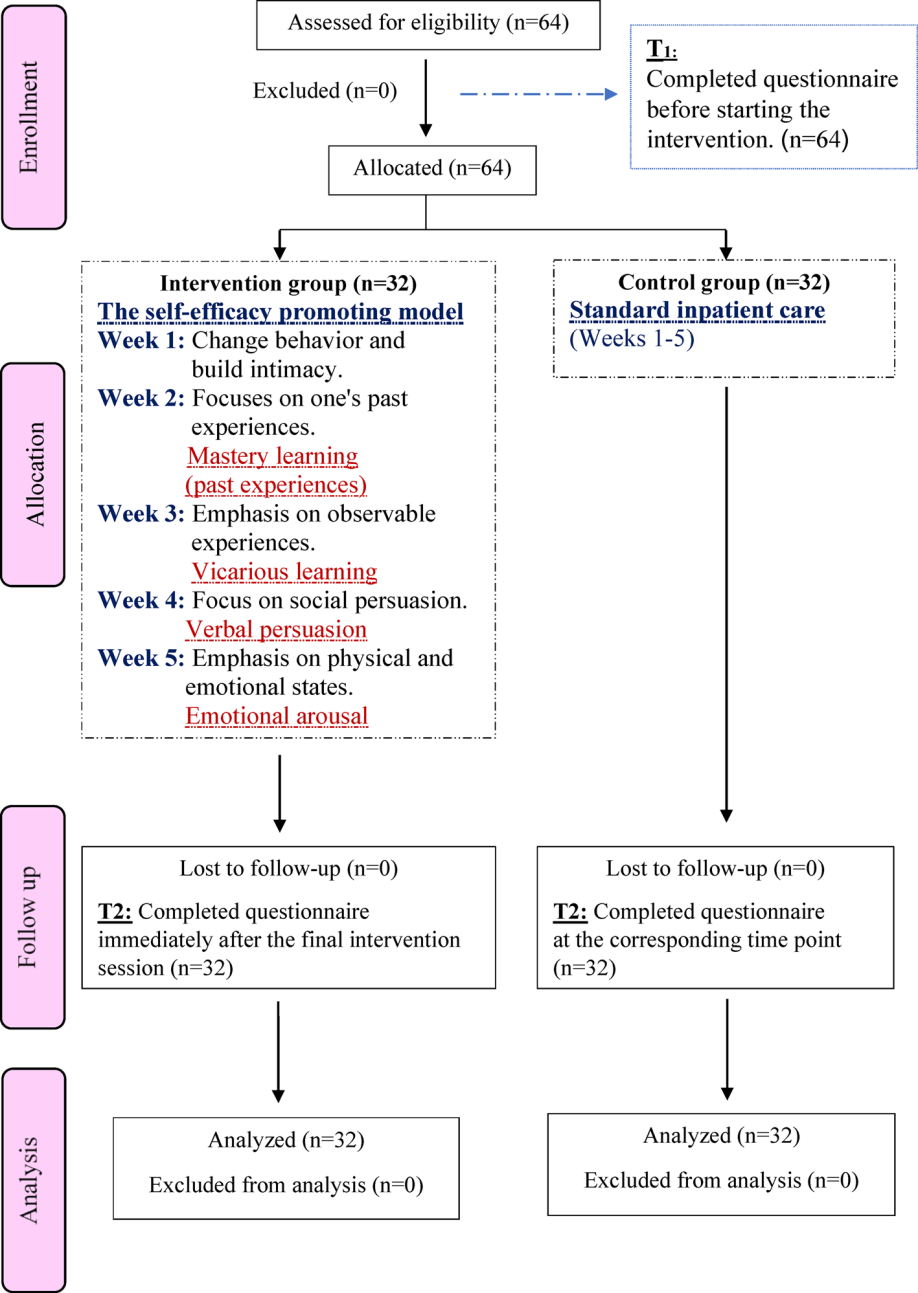
Flow diagram for the quasi-experimental study

This quasi-experimental research was designed evaluation conducted before the intervention (T1) and immediately following its conclusion (T2). Therefore, participants in both the intervention and control groups were asked to complete the post-assessment questionnaire immediately after the final session due to uncertainty regarding the discharge plan from the attending physician. The flow diagram of the study is in Fig. 2.

### Data analysis

Descriptive statistical analysis was performed using e.g. mean, standard deviation, percentage, and range. Baseline characteristics of participants in the intervention and control groups were compared using Chi-square test, whilst data across all four dimensions (Socrates-8 A, SADQ, AASE, BPRS) were compared using independent samples t-tests. Subsequently, an analysis was conducted by comparing scores before and after the intervention between the groups through paired samples t-tests. A significant level was set at 0.05.

## Results

The study included exclusively male participants aged 18–54 years, with a predominant proportion being single, having an educational attainment of grade nine or below, and belonging to the less skilled worker category. Among alcoholic patients diagnosed with schizophrenia, the length of illness ranged from 2 to 18 years, readmissions varied from one to eight times, and the onset of alcohol consumption occurred between the ages of 16 and 30 years. The study involved 64 participants, evenly divided into an intervention group and a control group of 32 males each, with no sample loss. As shown in Table 1, both groups were homogeneous in age, marital status, education level, and occupation, with no statistically significant differences (*p* > 0.05) according to the Chi-Square Test.

**Table 1:** Compare the differences in personal data between the intervention and control groups

Characteristics of participants	Intervention group *N* (percent)	Control group *N* (percent)	*x* ^2^	*P*-value
Gender				
Male	32 (100.0)	32 (100.0)		
Age (years)				
≤ 30	10 (31.3)	11 (34.4)	1.392	0.538
31–40	10 (31.3)	13 (40.6)		
>40	12 (37.4)	8 (25.0)		
Mean (SD) = 36.4 (9.6), Range = 21–54		Mean (SD) = 35.0 (7.8), Range = 18–48		
Marital status				
Single	19 (59.4)	13 (40.6)	2.375	0.305
Married	6 (18.7)	10 (31.3)		
Widowed	7 (21.9)	9 (28.1)		
Education				
≤ grade 9	14 (43.7)	20 (62.5)	2.259	0.510
>grade 9	18 (56.3)	12 (37.5)		
Occupation				
Less skilled workers	13 (40.6)	17 (53.1)	1.004	0.226
Skilled workers	19 (59.4)	15 (46.9)		

Note: SD, standard deviation

Before the intervention, the mean scores of the SOCRATES-8 A for AUD, SADQ, AASE, and BPRS did not differ significantly between the intervention and control groups (*p* > 0.05). Following the intervention, significant improvements were observed in the SOCRATES-8 A for AUD, AASE, and BPRS among participants in the intervention group compared with those in the control group (*p* < 0.05) (Table 2).

**Table 2:** Comparisons on outcomes of interest among participants in the intervention and control groups before and after the intervention using independent samples t-test

Before intervention				After intervention			
Intervention group Mean (SD)	Control group Mean (SD)	t	P-value	Intervention group Mean (SD)	Control group Mean (SD)	t	P-value
1. Stages of change readiness and treatment eagerness scale for alcohol use disorder							
50.66 (6.82)	50.56 (6.47)	0.06	0.955	81.44 (4.14)	52.44 (7.17)	19.81	< 0.001*
2. Severity of alcohol dependence questionnaire							
35.41 (9.18)	34.22 (7.36)	0.57	0.570	32.22 (8.43)	34.03 (6.66)	-0.96	0.343
3. Alcohol abstinence self-efficacy scale							
2.03 (0.31)	2.06 (0.25)	-0.36	0.724	4.27 (0.22)	2.08 (0.25)	37.19	< 0.001*
4. Brief psychiatric rating scale							
29.78 (2.45)	28.88 (2.47)	1.47	0.146	21.84 (1.30)	28.41 (2.59)	-12.82	< 0.001*

Note: SD, standard deviation, * *P* < 0.05

After the intervention concluded, it was observed that the readiness for change and enthusiasm for treatment (Socrates-8 A for AUD) significantly increased across all three areas: Recognition (56.31%), Ambivalence (59.19%), and Taking steps (65.69%). Similarly, there were notable improvements in self-efficacy for quitting alcohol (AASE), with increases in all four areas: Negative emotional effects and frustration (94.67%), Social pressure (173.38%), Pain associated with the body (69.77%), and Needs and motivation within oneself (133.52%). Demonstrated a statistically significant difference (p-value < 0.001). See Table 3.

**Table 3:** Comparison of the internal content of Socrates-8A for AUD and AASE, before and after intervention among participants in the intervention group (n = 32)

	Before		After		t-test	*P*-value
	Mean	SD	Mean	SD		
1. Recognition	19.25	2.81	30.09	1.55	18.05	< 0.001*
2. Ambivalence	10.66	1.77	16.97	1.15	16.41	< 0.001*
3. Taking step	20.75	3.17	34.38	2.45	19.28	< 0.001*
Total score	50.66	6.82	81.44	4.14	20.21	< 0.001*
Alcohol abstinence self-efficacy scale (AASE)						
1. Negative emotional effects and frustration	2.25	0.4	4.38	0.31	21.88	< 0.001*
2. Social pressure	1.54	0.4	4.21	0.27	39.82	< 0.001*
3. Pain associated with the body	2.58	0.59	4.38	0.32	13.33	< 0.001*
4. Needs and motivation within oneself	1.76	0.29	4.11	0.26	34.97	< 0.001*
Total score	2.03	0.31	4.27	0.22	32.11	< 0.001*

*Statistically significant at 0.05, SD, standard deviation; AASE, alcohol abstinence self-efficacy scale

Socrates-8A for AUD = stages of change readiness and treatment eagerness scale for alcohol use disorder

The overall paired t-test results, pairing changes in Socrates 8 A, AASE, and BPRS scores before and after the intervention program, demonstrated a statistically significant difference (p-value < 0.001). However, there were no statistically significant differences found in the change in SADQ scores, with a p-value of 0.206. See Table 4.

**Table 4:** A comparison of the Socrates 8A, SADQ, AASE, and BPRS assessments of participants before and after the alcohol use behavior modification program in schizophrenia patients in the intervention and control groups using paired samples t-test in the same group

Intervention group (n = 32)				Control group (n = 32)			
Pre-test mean (SD)	Post-test mean (SD)	t	*P*-value	Pre-test mean (SD)	Post-test mean (SD)	t	*P*-value
1. Stages of change readiness and treatment eagerness scale for alcohol use disorder							
50.66 (6.82)	81.44 (4.14)	20.21	< 0.001*	50.56 (6.47)	52.44 (7.71)	1.08	**0.289**
2 Severity of alcohol dependence questionnaire							
35.41 (9.18)	32.22 (8.43)	− 1.29	**0.206**	34.22 (7.36)	34.03 (6.66)	− 0.35	**0.731**
3 Alcohol abstinence self-efficacy scale							
2.03 (0.31)	4.27 (0.22)	32.11	< 0.001*	2.06 (0.25)	2.08 (0.25)	1.22	**0.232**
4 Brief psychiatric rating scale							
29.78 (2.45)	21.84 (1.30)	− 18.34	< 0.001*	28.88 (2.47)	28.41 (2.59)	− 1.49	**0.146**

*Statistically significant at 0.05, SD, standard deviation

## Discussion

In this study, we aimed to test the effectiveness of a self-efficacy-based intervention as an adjunctive treatment for modifying the behavior and thoughts associated with inappropriate alcohol use among alcoholic people admitted with schizophrenia, while also aiming to enhance the self-efficacy of the individuals involved. Over 5 weeks of treatment in a psychiatric hospital, which aligns with findings from a previous study [26]. This study assessed the prevalence of AUD and its determinants in schizophrenia patients admitted to a specialized psychiatric hospital in Addis Ababa, Ethiopia. Their sample included individuals over 18 years of age, with 414 randomly selected patients with schizophrenia. Their findings revealed a prevalence of AUD at 38.4%, with 22.4% categorized as Hazardous drinking (8–15 score’s AUDIT), 8.4% engaging in Harmful drinking (16–19) and Alcohol dependence (>20), and 7.6% exhibiting severe alcohol dependence. Factors associated with AUD included being male (Adjusted Odds Ratio; AOR = 5.8), single (AOR = 3.0), having a family history of alcoholism (AOR = 3.8), and experiencing prolonged illness duration (AOR = 3.9). This suggests that nearly four out of ten schizophrenia patients present with AUD, often characterized by being male, single, divorced or widowed, and having a family history of alcohol use, which is consistent with findings from Australia (38.0%) [27] and Switzerland (35.1%) [28].

The results of this program aimed at changing alcohol use behavior revealed a structured regimen consisting of 14 activities, each designed around the four key concepts of self-efficacy theory [11], with each session lasting between 60 and 90 min, conducted three times a week. The research findings indicated significant improvements: (1) The mean score for readiness to change regarding the treatment of alcohol drinking problems in the intervention group post-program; and (2) The mean scores for self-efficacy perception in quitting alcohol in the intervention group post-program were notably higher compared to pre-program. These findings are consistent with one previous study [29], which explored adjuvant treatment for AUD, demonstrating a positive impact on cognitive-behavioral modeling and self-efficacy. This quasi-experimental study, conducted in Turkey in 2015, involved 41 participants, with 20 individuals in the intervention group and 21 in the control group. The intervention group exhibited increased self-efficacy following program implementation, contributing to enhanced health outcomes.

When comparing the internal content of Socrates8A and AASE before and after implementing the program for changing alcohol use behavior, significant changes were observed in specific components within each scale using paired t-tests within the same group. These findings are consistent with a previous study, which investigated mindfulness-based interventions for individuals with substance use disorders based on cognitive self-efficacy theory [30]. Their research, involving 112 patients, demonstrated a significant increase in self-reliance scores from 76.50 ± 12.62 before the program to 85.50 ± 14.95 after the program, indicating the effectiveness of the intervention in enhancing self-efficacy.

In addition, findings from a study exploring the relationship between internalized stigma, self-efficacy, and motivation for treatment in individuals with substance abuse disorders, further support the importance of self-efficacy in treatment outcomes [31]. Their results highlighted a positive relationship between treatment motivation and self-efficacy levels, underscoring the significance of addressing internalized stigma to improve treatment outcomes and reduce substance use relapses.

In general, despite the demonstrated efficacy of various approaches in treating AUD, relapse remains a significant challenge, with relapse rates in the first-year post-treatment ranging from 60 to 90%. Interest in addressing relapse and other addictive disorders has intensified in recent years [32, 33]. Marlatt et al. [33] emphasized the importance of an individual’s coping mechanisms for situational triggers that may lead to relapse, along with addressing negative emotional reactions following an initial lapse. Crucially, individuals with effective coping strategies exhibit confidence in their ability to manage such situations, a key tenet of Bandura’s self-efficacy theory, which reduces the likelihood of relapse. Enhancing self-efficacy is therefore likely to lead to positive behavioral changes. Self-efficacy, in this context, refers to an individual’s belief in their capability to perform a behavior despite obstacles and to maintain control over their actions.

### Strengths and limitations

The research demonstrates strengths in its quasi-experimental design tailored to alcoholic people admitted with schizophrenia. The main researcher and co-researcher had comprehensive training in therapeutic modalities. In addition, the emphasis on self-efficacy enhancement throughout the intervention promotes active engagement and behavior change efforts among participants across the 14 activities in a 5-week program duration. However, a notable weakness lies in the short follow-up period, hindering a thorough understanding of sustained behavior changes and the prevention of re-hospitalization. In addition, physiological dependence on alcohol, as reflected by the SADQ, was found to be complex and showed no change during the follow-up period. Extending the follow-up to 3 months, 6 months, and 1 year would address this limitation. Moreover, the exclusive use of quantitative methods limits insights into participants’ post-program feelings and attitudes. Therefore, integrating qualitative research to capture holistic perspectives is imperative for a comprehensive understanding of the intervention’s impact; this should be explored in future research. Although there were no differences in participant characteristics or pre-assessment outcomes between the intervention and control groups, and all participants were recruited from the same hospital, selection bias may still have been presented. A randomized controlled trial could help confirm the efficacy of the program and reduce this bias. In addition, detailed data on individual drug types and dosages were not systematically collected, which may have affected the BPRS evaluation. Future studies should consider reporting specific antipsychotic regimens to strengthen the interpretation of outcomes.

## Conclusions

The findings suggest that this modification program could serve as an alternative intervention for alcoholic people admitted with schizophrenia. By fostering confidence in each individual and empowering them to exhibit behaviors conducive to goal attainment, such as reducing alcohol consumption, the program demonstrated a significant increase in Socrates 8 A and AASE scores, along with a decrease in BPRS scores, before and after the intervention. Based on the program’s success, policymakers should provide training for mental health professionals to manage and enhance self-efficacy among alcoholic patients with schizophrenia. A clinical practice guideline for treating the population also needs to be developed.

## Data Availability

Datasets are available on reasonable request to the corresponding author.

## Abbreviations

AASE: Alcohol abstinence self-efficacy scale
AUD: Alcohol use disorder
AUDIT: Alcohol use disorders identification test
AWS: Alcohol withdrawal scale
BPRS: Brief psychiatric rating scale
ICD-10: International classification of diseases, tenth revision
IOC: Index of item objective congruence
SADQ: Severity of alcohol dependence questionnaire
SEMASchizo program: Self-efficacy modification program among alcoholic people admitted with Schizophrenia
Socrates-8A: Stages of change readiness and treatment eagerness scale
TMSE: Thai Mental State Examination
WHO: World Health Organization
